# Serum 20S proteasome levels are associated with disease activity in MPO-ANCA-associated microscopic polyangiitis

**DOI:** 10.1186/s41927-020-00137-4

**Published:** 2020-08-25

**Authors:** Hiroshi Maruyama, Kouichi Hirayama, Marina Yamashita, Kentaro Ohgi, Ryuji Tsujimoto, Mamiko Takayasu, Homare Shimohata, Masaki Kobayashi

**Affiliations:** 1 Department of Nephrology, Tokyo Medical University Ibaraki Medical Center, 3-20-1 Chuo, Ami, Ibaraki, 300-0395 Japan; 2 Department of Intensive Care Medicine, Tokyo Medical University Ibaraki Medical Center, Ami, Ibaraki, Japan; 3 Department of Nephrology, Tokyo Medical University, Shinjuku, Tokyo, Japan

**Keywords:** ANCA-associated vasculitis, 20S-proteasome, Disease activity, Microscopic polyangiitis, Proteasome

## Abstract

**Background:**

Proteasomes are found in both the cell nucleus and cytoplasm and play a major role in the ubiquitin-dependent and -independent non-lysosomal pathways of intracellular protein degradation. Proteasomes are also involved in the turnover of various regulatory proteins, antigen processing, cell differentiation, and apoptosis. To determine the diagnostic value of serum proteasome in antineutrophil cytoplasmic antibody (ANCA)-associated vasculitis (AAV), we investigated patients with AAV at various stages of the disease.

**Methods:**

Serum 20S-proteasome was measured by ELISA in 44 patients with MPO-ANCA-associated microscopic polyangiitis (MPA) and renal involvement. Thirty of the patients provided serum samples before the initial treatment, and 30 provided samples during remission; 16 provided samples at both time points.

**Results:**

The mean serum 20S-proteasome level was significantly higher in the active-vasculitis patients (3414.6 ± 2738.9 ng/mL; *n* = 30) compared to the inactive-vasculitis patients (366.4 ± 128.4 ng/mL; *n* = 30; *p* <  0.0001) and 40 controls (234.9 ± 90.1 ng/mL; *p* <  0.0001). There were significant positive correlations between the serum 20S-proteasome level and the Birmingham Vasculitis Activity Score (BVAS) (*r* = 0.581, *p* <  0.0001), the ANCA titer (*r* = 0.384, *p* <  0.0001), the white blood cell (WBC) count (*r* = 0.284, *p* = 0.0042), the platelet count (*r* = 0.369, *p* = 0.0002), and the serum C-reactive protein (CRP) level (*r* = 0.550, *p* < 0.0001). There were significant negative correlations between the serum 20S-proteasome level and both the hemoglobin concentration (*r* = − 0.351, *p* = 0.0003) and the serum albumin level (*r* = − 0.460, *p* < 0.0001). In a multiple regression analysis, there was a significant positive correlation between the serum 20S-proteasome level and only the BVAS results (*β* = 0.851, *p* = 0.0009). In a receiver operating curve analysis, the area under the curve for the serum 20S-proteasome level was 0.996, which is higher than those of the WBC count (0.738) and the serum CRP level (0.963).

**Conclusion:**

The serum level of 20S-proteasome may be a useful marker for disease activity in AAV.

## Background

Proteasomes are located in both the nucleus and cytoplasm of cells, and they play a major role in the ubiquitin-dependent and ubiquitin-independent non-lysosomal pathways of intracellular protein degradation [1, 2]. Proteasomes are also involved in the turnover of various regulatory proteins (e.g., rate-limiting enzymes [3] and proteins for cell-cycle control [4] or transcriptional regulation [5]), antigen processing [6], cell differentiation [7], and apoptosis [8]. The 26S proteasome is a multicatalytic enzyme with a highly ordered structure composed of at least 32 different subunits arranged in two subcomplexes: a 20S core and a 19S regulator particle [9]. The 20S-proteasome is composed of four rings of 28 non-identical subunits (two rings are composed of seven alpha subunits, and the other two rings are composed of seven beta subunits). Three of the seven beta subunits have proteolytic sites; the β1, β2, and β5 subunits are associated with caspase-like, trypsin-like, and chymotrypsin-like activities, respectively [10]. These β1, β2, and β5 subunits cleave peptide bonds at post-acidic, −basic, and -hydrophobic amino acid residues, respectively [10]. However, subunits β1, β2, and β5 could be replaced with β1i, β2i, and β5i by interferon-gamma (IFN-γ), and this IFN-γ-inducible proteasome isotype is called the immunoproteasome [11].

The serum proteasome levels of patients with malignant tumors are elevated because the proteasome is overexpressed in tumor cells. In patients with multiple myeloma, serum proteasome concentrations have been shown to be associated with disease severity and activity [12]: the serum proteasome concentrations were significantly elevated in patients with multiple myeloma compared to controls, in multiple myeloma versus monoclonal gammopathies of undetermined significance (MGUS), and in active versus smoldering multiple myeloma [12]. Similarly, elevated serum proteasome levels were also reported in autoimmune diseases characterized by B-cell abnormality [13]. In the present study, to determine the diagnostic value of the serum proteasome concentration in antineutrophil cytoplasmic antibody (ANCA)-associated vasculitis (AAV), we investigated patients with myeloperoxidase (MPO)-AAV at various stages of the disease.

### Patients

#### Patients and controls

We analyzed the cases of 44 patients with MPO-ANCA-associated microscopic polyangiitis (MPA) and renal involvement. The diagnosis of MPA was based on the European Medicines Agency algorithm [14], and patients with other types of systemic vasculitis (including eosinophlic granuromatosis with polyangiitis, granulomatosis with polyangiitis, and anti-glomerular basement disease) were excluded.

Of the 44 MPO-AAV patients, 30 provided serum samples before the initial treatment, and 30 provided samples during remission; 16 provided samples both before the initial treatment and during remission. The Birmingham Vasculitis Activity Score (BVAS) was used to evaluate patients’ disease activity, and remission was defined as a BVAS of 0. As controls, 14 healthy volunteers and 26 patients with chronic kidney disease (CKD) were investigated. The causes of CKD were nephrosclerosis (*n* = 10), chronic glomerulonephritis (*n* = 11), diabetic nephrosclerosis (*n* = 3), and autosomal dominant polycystic kidney disease (*n* = 2).

#### Sample collection and analysis

The serum samples measured by a commercially available enzyme-linked immunosorbent assay (ELISA) kit (Enzo Life Science, Plymouth Meeting, PA, U.S.) in duplicate. In brief, 96-well microtiter plates were coated with a mouse anti-20S-proteasome alpha-6 subunit monoclonal antibody and left overnight at 4 °C, followed by blocking with phosphate-buffered saline (PBS) containing bovine serum albumin for 2 h at room temperature (RT). A serum sample was then added to each well, and the plates were incubated for 1 h at RT. A rabbit anti-20S-proteasome polyclonal antibody was then added to each well, and the plates were incubated for 1 h at RT, followed by incubation with a horseradish-peroxidase-labeled goat anti-rabbit IgG antibody for 1 h at RT. The plates were finally incubated with chromogen (tetramethylbenzidine) and hydrogen peroxide for 30 min at RT and then added with 1 N hydrochloride acid solution.

Between these steps, the plates were washed five times with Tris-buffered saline. The plates were immediately read on a microplate reader (Sunrise Remote®, Tecan Japan, Kanagawa, Japan) set at 450 nm with 540 nm as a reference wavelength. The inter- and intra-assay variations were < 10%.

#### Statistical analyses

All statistical analyses were performed using PASW Statistics software, ver. 18 (IBM Japan, Tokyo) for Windows. The data are expressed as means ± standard deviations or as numbers with percentages of the total. The chi-square test with Yates’ continuity correction and Fisher’s exact test were used for differences in categorical variables, and post-hoc comparisons (Bonferroni correction) were performed to detect differences among three groups. The Mann-Whitney U-test was used for two-group comparisons, and we conducted an analysis of variance (ANOVA) to assess differences among three or more groups; post-hoc comparisons were made using the Bonferroni/Dunn test. Correlations were determined using Spearman’s univariate correlation test and a linear regression analysis. The multiple linear regression analysis included the covariates shown to be significantly associated with the serum 20S-proteasome level in the correlation analysis, and the data are expressed as standardized regression coefficients (*β*). We applied comparative receiver-operating-characteristic (ROC) curves and the area under the curve (AUC) to assess the disease activity accuracy of the the serum 20S-proteasome level and inflammatory variables. *P*-values were accepted as significant at < 0.05, but in the comparisons of three or more groups, the critical *p*-value was divided by the number of comparisons being made.

## Results

### The subjects’ characteristics

The characteristics, clinical symptoms and laboratory data among the subjects of the three groups (the active-vasculitis patients, the inactive-vasculitis patients, and the controls) are shown in Table 1. At the testing, there was no patients treated with any immunosuppressant in both active and inactive vasculitis, but all inactive-vasculitis patients had treated with corticosteroids (doses of prednisolone, 5.00 ± 1.97 mg/day).

**Table 1:** Characteristics of subjects

	MPO-ANCA associated vasculitis		Controls (*n* = 40)
	Active-vasculitis (*n* = 30)	Inactive-vasculitis (*n* = 30)	
Age (years)	71.4 ± 14.2	69.7 ± 13.2	69.3 ± 12.0
Gender (male:female)	17: 13	17: 13	21: 19
Birmingham vasculitis activity score	20.7 ± 5.2^*, **^	0 ± 0	
Clinical symptoms			
Fever	21 (70%)^*^	0 (0%)	
Weight loss	10 (33%)^*^	0 (0%)	
Arthralgia	22 (73%)^*^	0 (0%)	
Episcleritis or uvitis	2 (7%)	0 (0%)	
Sinusitis	1 (3%)	0 (0%)	
Hearing loss	3 (10%)	0 (0%)	
Alveolar hemorrhage	5 (17%)	0 (0%)	
Interstitial lung disease	15 (50%)	13 (43%)	
Arrhythmia	3 (10%)	0 (0%)	
Pericarditis	2 (7%)	0 (0%)	
Heart failure	9 (30%)^*^	0 (0%)	
Rapidly progressive glomerulonephritis	25 (83%)^*^	0 (0%)	
Peripheral nerve damage	1 (3%)	0 (0%)	
Laboratory data			
ANCA titer (U/mL)	255.7 ± 178.8^*, **^	14.2 ± 30.4	
White blood cell (/mm^3^)	9483 ± 3380^*, **^	8923 ± 3234^**^	5763 ± 1482
Hemoglobin conc. (g/dL)	9.1 ± 2.0^*, **^	11.7 ± 1.8	12.1 ± 2.0
Platelet count (10^4^/mm^3^)	27.2 ± 12.0^*, **^	21.2 ± 5.4	20.2 ± 6.0
Serum albumin (g/dL)	3.05 ± 0.60^*, **^	3.74 ± 0.37^**^	4.10 ± 0.34
Serum creatinine (mg/dL)	4.55 ± 3.33^*, **^	3.26 ± 3.96	1.76 ± 1.48
Serum C-reactive protein (mg/dL)	7.92 ± 6.87^*, **^	0.18 ± 0.16	0.09 ± 0.12
Serum 20S-proteasome (mg/dL)	3414.6 ± 2738.9^*, **^	366.4 ± 128.4	234.9 ± 90.1
Doses of prednisolone (mg daily)	0 ± 0^*^	5.00 ± 1.97	

^*^P < 0.0167 vs. Inactive-vasculitis; ^**^P < 0.0167 vs. Controls

### Serum 20S-proteasome levels

**Fig. 1:**
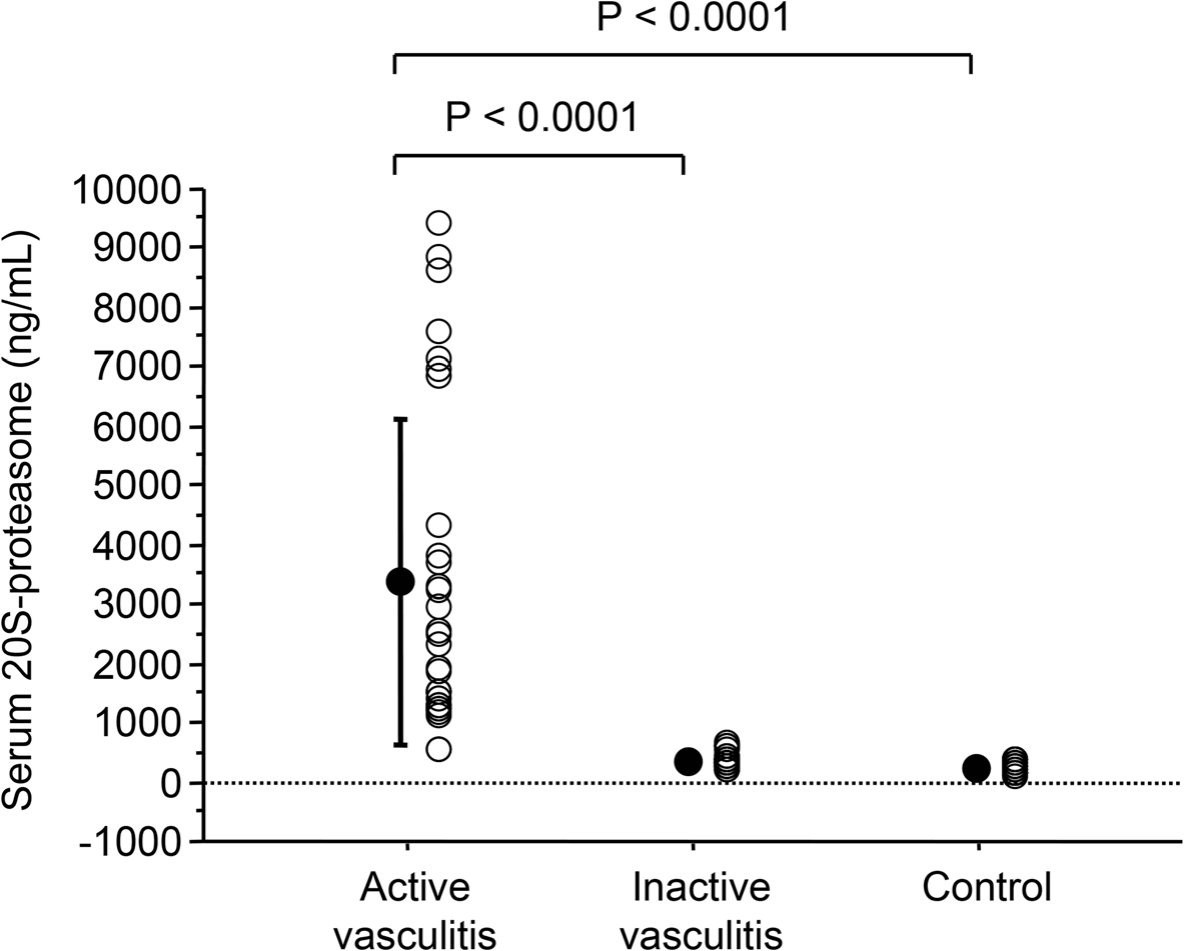
The serum levels of 20S-proteasome. *Closed circles* = means, *bars* = standard deviations. *Open circles:* the values for individual patients

As illustrated in Fig. 1, the mean level of serum proteasome in the active-vasculitis patients (3414.6 ± 2738.9 ng/mL) was significantly higher than those in the inactive-vasculitis patients (366.4 ± 128.4 ng/mL; *p* < 0.0001) and the controls (234.9 ± 90.1 ng/mL; *p* < 0.0001). There were significant positive correlations between the serum 20S-proteasome levels and the BVAS results (*r* = 0.581, *p* < 0.0001), the MPO-ANCA titers (*r* = 0.384, *p* < 0.0001), the WBC counts (*r* = 0.284, *p* = 0.0042), the platelet counts (*r* = 0.369, *p* = 0.0002), and the serum CRP levels (*r* = 0.550, *p* < 0.0001). There were significant negative correlations between the serum 20S-proteasome levels and both the hemoglobin concentrations (*r* = − 0.351, *p* = 0.0003) and the serum albumin levels (*r* = − 0.460, *p* < 0.0001). In the multiple regression analysis, there was a significant positive correlation between the serum 20S-proteasome levels and only the BVAS results (*β* = 0.851, *p* = 0.0009, Table 2).

**Table 2:** Correlation between the serum 20S-proteasome level and clinical parameters

	Univariate analysis		Multivariate analysis	
	*r*	*P*-value	*β*	*P*-value
Age	0.047	0.6424	–	–
Birmingham Vasculitis Activity Score	0.581	< 0.0001	0.851	0.0009
ANCA titer	0.384	< 0.0001	−0.335	0.0523
White blood cell	0.284	0.0042	−0.137	0.3068
Hemoglobin conc.	−0.351	0.0003	0.153	0.3479
Platelet count	0.369	0.0002	0.220	0.0938
Serum albumin	−0.460	< 0.0001	−0.137	0.8223
Serum creatinine	0.153	0.1282	–	–
Serum C-reactive protein	0.550	< 0.0001	−0.031	0.8756

In the active-vasculitis patients, there was no association between the serum 20S proteasome levels and clinical symptoms except for pulmonary involvement (Supplementary file S1). The mean serum 20S proteasome level in the active-vasculitis patients with interstitial lung disease (*n* = 15; 4994.3 ± 3025.8 ng/mL) was significantly higher than those in the active-vasculitis patients without pulmonary involvement (*n* = 10; 2110.0 ± 1114.0 ng/mL; *p* = 0.0045) and those in active-vasculitis patients with alveolar hemorrhage (*n* = 5; 1284.5 ± 515.9 ng/mL; *p* = 0.0040). There was no association between the serum 20S proteasome levels and the percentages of crescent formation, renal histological classification (Berden’s classification [15]), or renal symptoms (patients with chance proteinuria/hematuria and patients with rapidly progressive glomerulonephritis).

### The diagnostic potential for disease activity

**Fig. 2:**
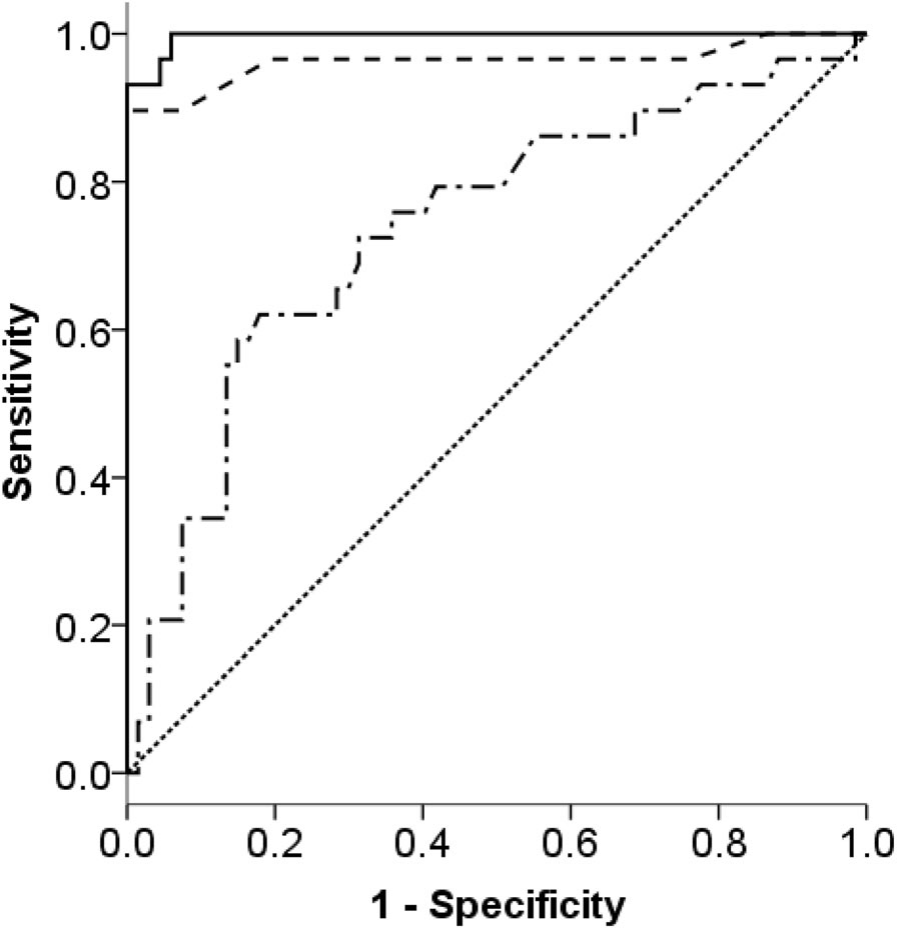
The comparative ROC curves for three measurements of disease activity. *Solid line:* serum levels of 20S-proteasome. *Dash-dotted line:* WBC counts. *Dashed line:* serum CRP levels. *Dotted line:* reference line

The optimum cut-off levels for the disease activity of vasculitis were identified from the ROC curves for the WBC count (> 7250/mm^3^), serum CRP level (> 0.72 mg/dL), and serum 20S-proteasome level (> 563.1 ng/mL) (Fig. 2). The area under the curve (AUC) for the serum 20S-proteasome level was 0.996, which is higher than those of the WBC count (0.738) and the serum CRP level (0.963). On the ROC curve, the serum 20S-proteasome had 96.6% sensitivity and 95.5% specificity for the disease activity. Although the specificity of the serum 20S-proteasome level was less than that of the serum CRP level (100%), the sensitivity of the serum 20S-proteasome level was superior to that of the serum CRP level (89.7%; Table 3).

**Table 3:** Comparative ROC curves for 3 parameters of disease activity

	Area under the curve	95% confidence interval	*P*-value	Optimal cut-off levels	Sensitivity (%)	Specificity (%)
White blood cell count	0.738	0.624–0.851	< 0.001	7250	72.4	68.7
Serum C-reactive protein	0.963	0.000–1.000	< 0.001	0.72	89.7	100
Serum 20S-proteasome	0.996	0.000–1.000	< 0.001	563.1	96.6	95.5

## Discussion

Previous studies have demonstrated that the serum-20S-proteasome levels are elevated in individuals with autoimmune diseases. In 314 patients with various autoimmune diseases including systemic lupus erythematosus (SLE), polymyositis, Sjögren’s syndrome, antiphospholipid syndrome, rheumatoid arthritis (RA), systemic sclerosis, autoimmune hepatitis, and myasthenia gravis, the serum proteasome levels were higher than in the 85 controls [13]. The levels were especially (and significantly) high in the patients with SLE, polymyositis, Sjögren’s syndrome, RA, and autoimmune hepatitis [13]. In patients with polymyositis, serum proteasome levels were correlated with serum creatinine kinase levels, and serum proteasome levels were associated with disease activity [13]. In the present study, elevated serum 20S-proteasome levels were also demonstrated in patients with AAV. Although there was no relationship between the MPO-ANCA titers and the serum 20S-proteasome levels, these elevations were associated with disease activity (i.e., the BVAS). Therefore, the serum level of 20S-proteasome may be a useful marker for disease activity in AAV.

The mechanisms that underlie the elevated serum proteasome observed in patients with AAV are not yet known. Several serum biomarkers are filtrated at the glomerulus and reabsorbed and catabolized by proximal tubular cells, and the serum levels of such biomarkers in patients with renal insufficiency are elevated due to low urinary filtration. Because we found no relationship between serum 20S-proteasome levels and serum creatinine in AAV patients, we conclude that elevated serum proteasome is not associated with renal function.

In a previous investigation, the serum proteasome levels in patients with multiple myeloma were elevated due to overexpression in tumor cells, but the mechanisms underlying these elevations in autoimmune diseases were not clarified [16]. On the other hand, the structure and function of serum proteasome in healthy donors and patients with autoimmune diseases (SLE and RA) were maintained in the same manner as the intracellular forms [16]. However, β-rings of proteasomes in the serum of patients with autoimmune diseases were different from those in healthy donors, and those rings contained immunosubunits β2i and β5i [16]. Considering that proteasomes from non-immunocompetent cells do not contain immunosubunits [17], it was speculated that serum proteasome in patients with autoimmune diseases may have its fraction structure added by an immunocompetent cell origin that is different from that in normal individuals. Therefore, the elevated serum proteasome levels in AAV may also be associated with the activation of immunocompetent cells. Further investigations are needed to clarify the mechanism by which the proteasome is released into the circulation.

Bortezomib, a proteasome inhibitor, prevents the degradation of proteins marked by ubiquitination by inhibiting the 26S proteasome [18]. The main effects of bortezomib are NF-κB inhibition, inhibition of cell proliferation by the stabilization of cyclin-dependent kinases, the induction of apoptosis by the activation of c-Jun-NH_2_ terminal kinase, the stabilization of proapoptotic proteins and transcription factors and tumor suppressors, and the induction of cell death by activation of the terminal unfolded protein response [19]. Bortezomib has been approved for clinical use in patients with multiple myeloma, and bortezomib treatment has implications for antibody-mediated immune diseases as well [20].

The efficacy of bortezomib was demonstrated in a mouse model of MPO-ANCA-associated glomerulonephritis [21]. That is, in anti-MPO-associated glomerulonephritis induced by immunizing MPO-deficient mice with murine MPO followed by irradiation and the transplantation of wild-type bone marrow, proteinuria (albuminuria) and hematuria were significantly reduced compared to the controls by both standard steroid/cyclophosphamide treatment and bortezomib treatment [22]. Moreover, the percentage of glomeruli with crescent or necrosis formation was reduced by both treatments. The clinical efficacy of bortezomib for AAV has not been determined, because only one case of an AAV patient treated with bortezomib was reported. In that case, complete remission could not be achieved by a combination treatment with corticosteroid, cyclophosphamide, and rituximab; therefore, bortezomib (1.3 mg/m^2^/week for 4 weeks) was added [22]. After the addition of bortezomib the patient achieved complete remission, and the doses of corticosteroid could be withdrawn [22]. Thus, the proteasome may be associated with the development of AAV, and inhibition of the proteasome may be effective for inducing the remission of AAV.

Our study has several limitations. The study population was small and limited to MPO-AAV patients with renal involvement, and thus further studies are needed to compare patients with PR3-AAV or non-renal vasculitis. In addition, this was a retrospective cross-sectional study; a larger prospective longitudinal study (including vasculitis patients with relapse) would provide more definitive results. Since the present study was performed at one facility, it is necessary to verify the accuracy of the ELISA test. Moreover, all inactive-vasculitis patients were treated with corticosteroids at the testing, so treatments themselves may affected to decreased levels in inactive vasculitis. Therefore, further studies are needed to compare AAV patients without treatments at the testing, or to investigate other diseases patients treated with/without corticosteroids. Finally, although we did demonstrate that serum 20S-proteasome levels were elevated in our patients with AAV, the cause of this elevation was not identified. In patients with multiple myeloma, elevation of serum 20S-proteasome may be associated with overexpression in tumor cells or abnormal cellular turnover [12]. On the other hand, elevation of serum 20S-proteasome was observed in septic patients and the relation between elevated serum 20S-proteasome levels and increased lymphocyte apoptosis was demonstrated in critically ill patients [23]. In patinets with RA and SLE, it was speculated that the expression of inflammatory cytokines may have influenced the elevation of the serum 20S-proteasome [13]. Althoug elevated serum 20S-proteasome in active AAV may be reflected an acute phase response, there was no significant correlation between the serum 20S-proteasome levels and serum CRP levels in the multiple regression analysis. To clarify the mechanisms of the serum 20S-proteasome elevation in vasculitis patients, further in vitro and ex vivo investigations are needed.

## Conclusion

The serum levels of 20S-proteasome in our patients with active MPO-AAV were significantly elevated compared to the levels in the patients with inactive MPO-AAV and the controls. Moreover, the serum levels of 20S-proteasome were related to the BVAS results. The serum level of 20S-proteasome may therefore be a useful marker for disease activity in AAV.

## Supplementary information

**Additional file 1: Supplementary file S1.** The relationships between the serum 20S proteasome levels and the patients’ clinical symptoms.

## Data Availability

All of the raw datasets used and analyzed in this study are available upon reasonable request from the corresponding author.

## Abbreviations

AAV: Antineutrophil cytoplasmic antibody-associated vasculitis
ANCA: Antineutrophil cytoplasmic antibody
BVAS: Birmingham Vasculitis Activity Score
MPA: Microscopic polyangiitis
MPO: Myeloperoxidase
