# Supplementary material for: Serum 20S proteasome levels are associated with disease activity in MPO-ANCA-associated microscopic polyangiitis
Source: BMC Rheumatol. 2020 Aug 25;4:36. doi: 10.1186/s41927-020-00137-4 (PMC7447580; doi:10.1186/s41927-020-00137-4)
Supplement: Supplementary file 1 — Additional file 1: Supplementary file S1. The relationships between the serum 20S proteasome levels and the patients’ clinical symptoms. [file 41927_2020_137_MOESM1_ESM.docx]

# **Supplementary file 1.** The relationships between the serum 20S proteasome levels and the patients' clinical symptoms.

| characteristics | | No.of  patients | serum 20S-proteasome | | | P-value |
| --- | --- | --- | --- | --- | --- | --- |
| fever | absent | (n = 9) | 3474 | ± | 3005 | 0.9459 |
|  | present | (n = 21) | 3389 | ± | 2695 |  |
| weight loss | absent | (n = 20) | 3713 | ± | 3062 | 0.8259 |
|  | present | (n = 10) | 2818 | ± | 1946 |  |
| arthralgia | absent | (n = 8) | 3371 | ± | 3195 | 0.6730 |
|  | present | (n = 22) | 3430 | ± | 2637 |  |
| episcleritis / uveitis | absent | (n = 28) | 3549 | ± | 2788 | 0.5060 |
|  | present | (n = 2) | 1526 | ± | 27 |  |
| sinusitis | absent | (n = 29) | 3513 | ± | 2733 | 0.1188 |
|  | present | (n = 1) | 568 |  |  |  |
| hearing loss | absent | (n = 27) | 3459 | ± | 2883 | 0.6041 |
|  | present | (n = 3) | 3013 | ± | 692 |  |
| pulmonary involvement | absent | (n = 10) | 2110 | ± | 1114 | 0.0106 |
|  | DAH | (n = 5) | 1284 | ± | 516 |  |
|  | ILD | (n = 15) | 4994 | ± | 3026 |  |
| arrhythmia | absent | (n = 27) | 3260 | ± | 2602 | 0.4266 |
|  | present | (n = 3) | 4805 | ± | 4186 |  |
| pericarditis | absent | (n = 28) | 3294 | ± | 2626 | 0.4543 |
|  | present | (n = 2) | 5098 | ± | 5024 |  |
| heart failure | absent | (n = 21) | 3949 | ± | 2901 | 0.0738 |
|  | present | (n = 9) | 2168 | ± | 1910 |  |
| rapidly progressive glomerulonephritis | absent | (n = 5) | 3694 | ± | 2403 | 0.4867 |
|  | present | (n = 25) | 3359 | ± | 2843 |  |
| peripheral nerve injury | absent | (n = 29) | 3234 | ± | 2599 | 0.1487 |
|  | present | (n = 1) | 8651 |  |  |  |
| Renal histological classification | focal | (n = 5) | 3740 | ± | 2323 | 0.6641 |
|  | cellular | (n = 5) | 4011 | ± | 2878 |  |
|  | mixed | (n = 3) | 3165 | ± | 3176 |  |
|  | sclerotic | (n = 1) | 2476 |  |  |  |
